# Long-Term Management of Basal Cell Carcinoma Undergoing Reconstructive Procedures Before the Acquisition of Negative Margins

**DOI:** 10.7759/cureus.91538

**Published:** 2025-09-03

**Authors:** Ismail H Unal, Aysenur Botsali, Semanur Dogan, Ercan Caliskan

**Affiliations:** 1 Dermatology, University of Health Sciences Gülhane Training and Research Hospital, Ankara, TUR

**Keywords:** basal cell carcinoma (bcc), long term management, surgery, treatment choices, vismodegib

## Abstract

Reconstructive surgery without achieving negative margins in basal cell carcinoma (BCC) increases the risk of recurrence. We present a case of a 68-year-old male patient diagnosed with BCC on the left cheek who underwent reconstruction without achieving negative surgical margins. The patient experienced postoperative recurrence, and the lesions recurred despite various treatments. We have followed this patient for approximately 20 years with multiple treatment modalities, and his case highlights the challenges of long-term management associated with reconstruction without achieving negative margins and the importance of combined treatment strategies.

## Introduction

Basal cell carcinoma (BCC) is the most common malignant tumor [1]. Given that metastasis is exceedingly rare, BCC typically does not compromise patient survival expectancy [1,2]. Although BCC rarely metastasizes, its capacity for local invasion may lead to significant tissue destruction, especially when occurring in high-risk anatomical areas such as the central face, periocular region, or scalp [2]. Incomplete excision in these locations may result in recurrence, larger surgical defects, and greater morbidity.

The standard of care remains surgical excision with histologically confirmed clear margins [3]. Achieving negative margins is critical, as the risk of recurrence increases significantly when residual tumor persists [4]. Risk assessment should assess for tumor location, histopathologic subtype, and clinical characteristics, as these variables are closely linked to recurrence rates. Facial BCCs are classified as high-risk owing to the region’s anatomical intricacy and heightened cosmetic relevance [5]. In these situations, treatment should be planned to ensure effective tumor control while minimizing functional and aesthetic compromise.

Although surgery remains the cornerstone of treatment, not all patients are ideal candidates for extensive excision or complex reconstruction. For these individuals, alternative therapies such as radiotherapy, topical agents (e.g., imiquimod, 5-fluorouracil), or systemic Hedgehog pathway inhibitors like vismodegib may be considered [6]. Vismodegib has demonstrated efficacy in advanced or recurrent BCC but is limited by side effects such as dysgeusia and muscle cramps [7]. Topical treatments, while typically reserved for superficial or low-risk lesions, may be used in selected patients as part of a multimodal strategy under close clinical monitoring [8].

Performing reconstruction before histologic confirmation of clear margins, particularly in high-risk facial tumors, can lead to persistent or multifocal recurrence and complicate subsequent management [4,5]. Managing such complex cases typically involves a prolonged, multimodal approach that integrates surgical intervention, topical and systemic therapies, and close clinical and dermoscopic follow-up. We present a case involving a rare instance of recurrent BCC of the cheek, managed over nearly two decades following flap reconstruction without prior margin clearance. This case demonstrates the complexity of managing high-risk recurrent BCC and emphasizes the role of coordinated, multidisciplinary care supported by careful oncologic assessment during both surgical planning and long-term monitoring.

Informed consent for publication of identifiable clinical images was obtained from the patient and submitted to the journal.

## Case presentation

A 68-year-old male patient initially presented to our outpatient clinic in 2016, exhibiting multiple pigmented lesions along the margins of a prior scar on his left cheek. His medical history was notable for BCC localized to the left cheek, diagnosed in 2006. The lesion had initially manifested as a crusted wound and was subsequently identified as a nodular BCC, which had been managed with two excisional surgeries. However, recurrences had prompted a third surgical excision accompanied by flap reconstruction. Despite these interventions, the lesion had recurred post-reconstruction, leading to the administration of radiotherapy in 2011.

**Figure 1:**
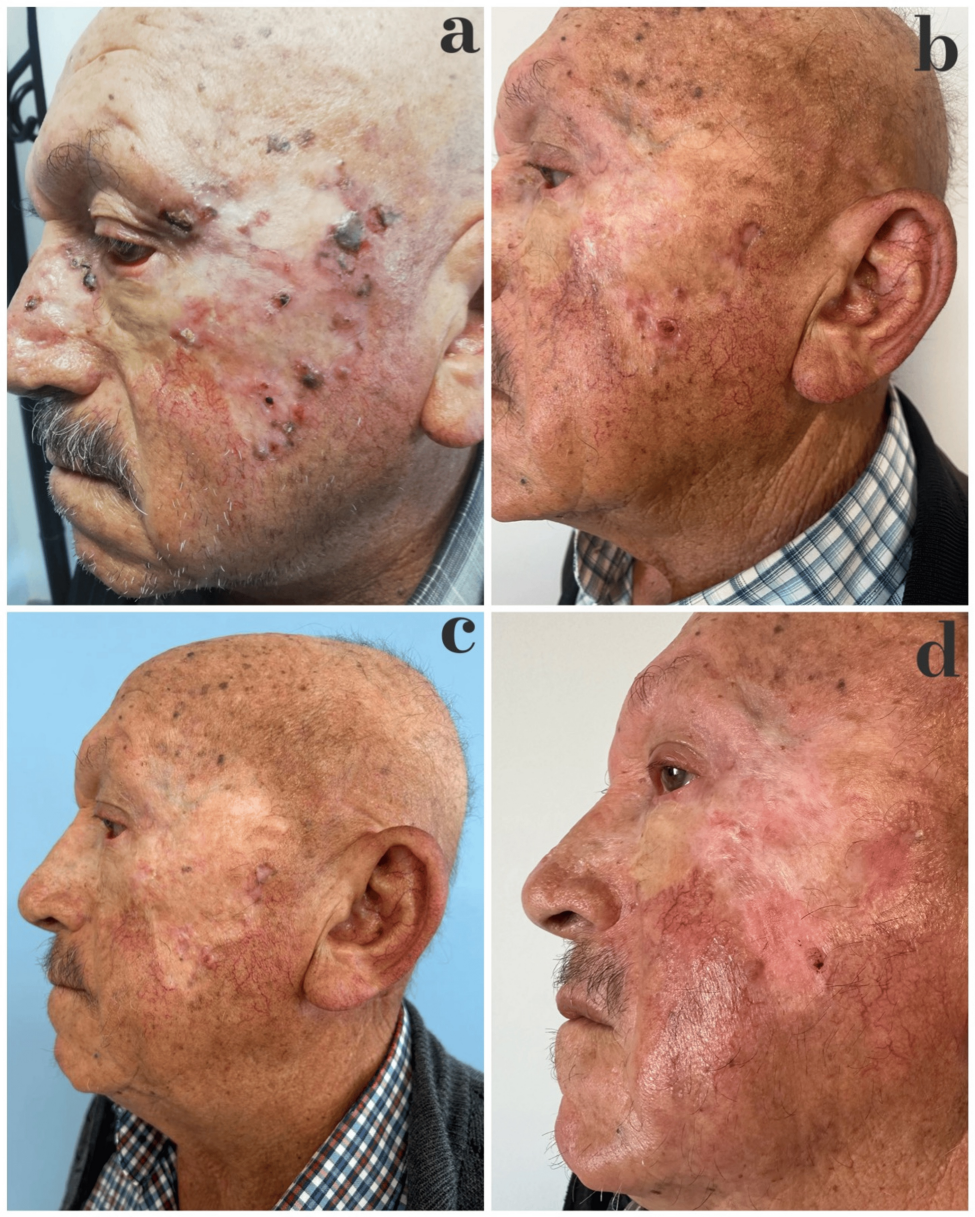
Lesion progression during treatment (2021–2024) Clinical progression of the lesion under treatment. (a) Before vismodegib treatment (2021), (b) after 12 months of vismodegib (2022), (c) five months after topical 5-fluorouracil (2023), (d) during combined cryotherapy and imiquimod treatment (2024)

**Figure 2:**
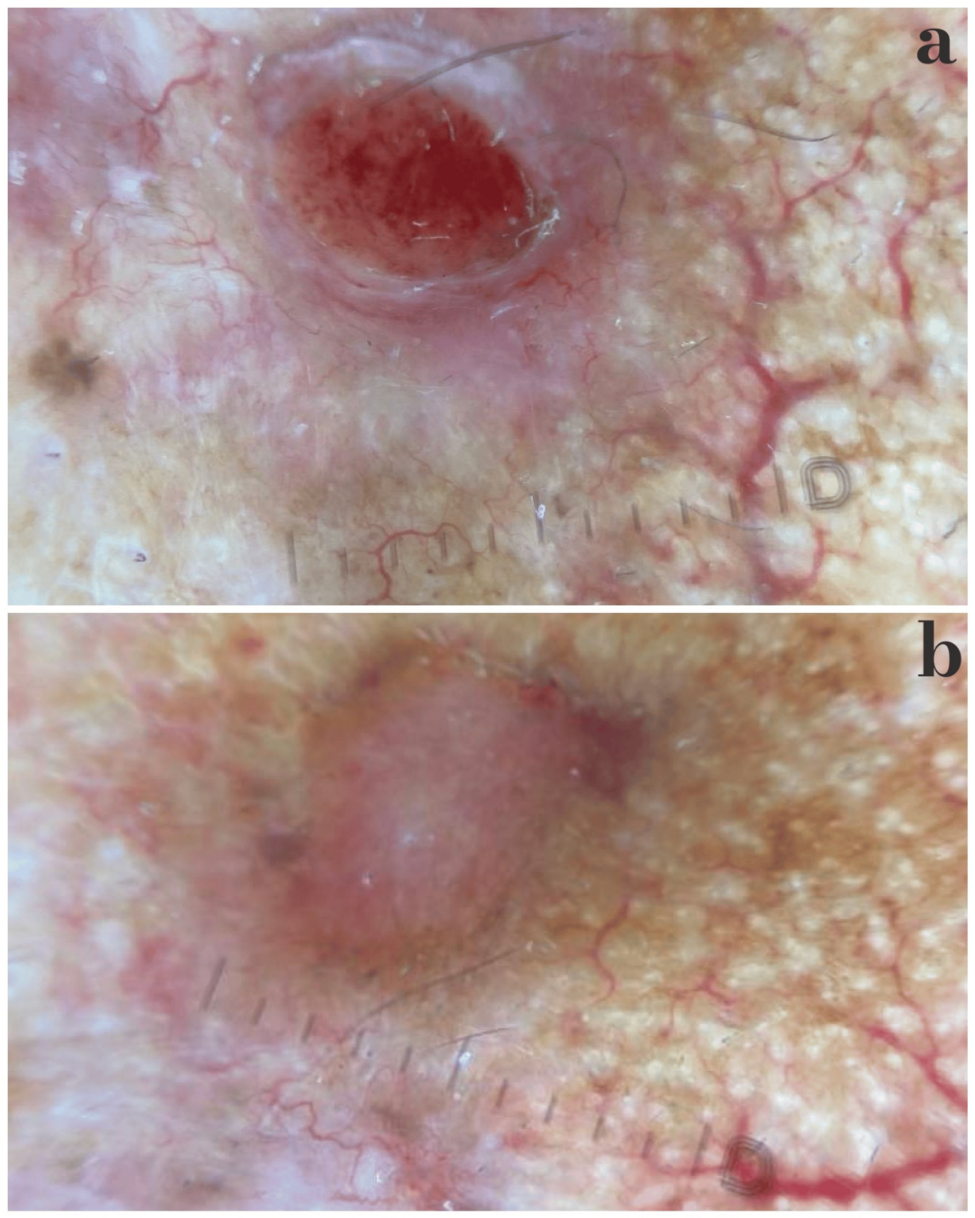
Dermoscopic views of the patient’s basal cell carcinoma lesions Dermoscopic views of basal cell carcinoma. (a) Central ulceration/erosion with arborizing vessels of irregular caliber, occasional coiled segments, scattered grey–brown dots and blotches, and small whitish structureless areas. (b) Well-circumscribed pink nodule with arborizing vessels (thick trunks and thin irregular branches), fine telangiectasias, focal grey–brown dots, structureless pigmentation, and whitish areas

In 2016, the patient was initiated on vismodegib at a dosage of 150 mg daily - an oral Hedgehog pathway inhibitor - resulting in complete remission after six months of treatment. Following this positive response, the patient was maintained under observation without further therapeutic interventions. However, in 2021, multiple pigmented lesions were identified along the margins of the scar on the left cheek, evaluated as a recurrence (Figure 1a). Clinical examination revealed mild ectropion of the left lower eyelid related to previous interventions. Dermoscopic evaluation showed features consistent with BCC (Figure 2).

Vismodegib therapy was reinitiated at a dosage of 150 mg per day. The patient showed a marked but incomplete response, with a reduction in lesion size, decreased pigmentation, and partial regression of dermoscopic vascular features (Figure 1b). Clinical and dermoscopic examination revealed a persistent nodular component with atypical arborizing vessels and residual pigmentation, consistent with residual BCC. The treatment was continued for a duration of 12 months. The medication was discontinued upon the patient's request due to the development of taste disturbance, a common side effect of vismodegib treatment. The patient was treated with topical 5-fluorouracil 5% cream applied twice daily. Although an initial partial response was observed, recurrences and resistance became evident during follow-up. Consequently, the treatment was discontinued after approximately five months (Figure 1c).

**Figure 3:**
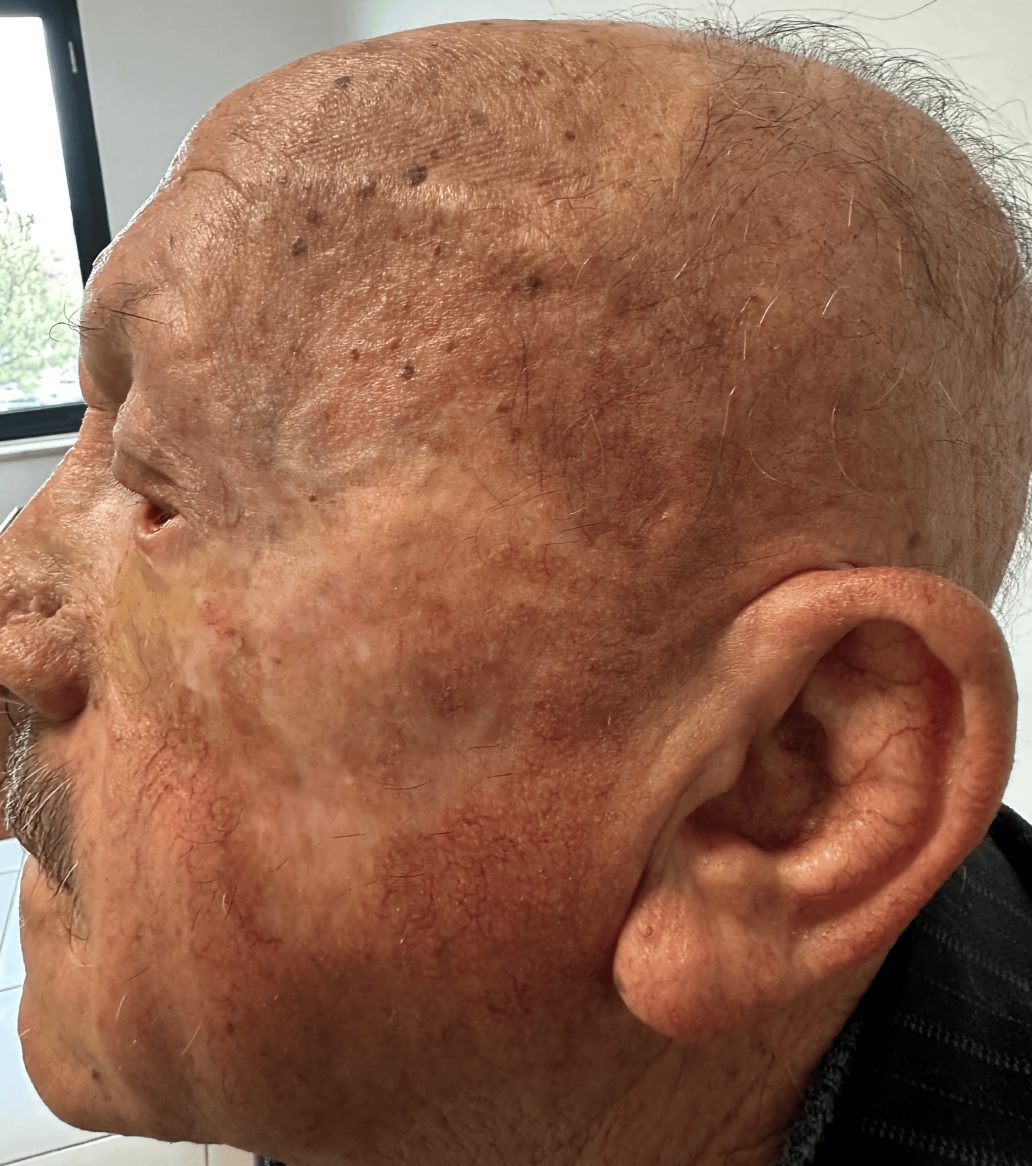
Clinical outcome after therapy (2025) Post-treatment presentation (2025) demonstrating complete clearance of the lesion following combined cryotherapy and topical imiquimod therapy. No residual clinical or dermoscopic signs of basal cell carcinoma are observed

A combination regimen of cryotherapy and topical imiquimod 5% cream was then initiated. Cryotherapy was selectively applied to clinically visible nodular components in three sessions at two-week intervals using liquid nitrogen, with two freeze-thaw cycles of approximately 20-30 seconds each, aiming at adequate tissue necrosis. Imiquimod was started the day after each cryotherapy session and applied once daily, five days per week; treatment was continued thereafter until the last follow-up. Mild local irritation occurred but was well tolerated. This regimen resulted in complete clinical and dermoscopic clearance (Figure 1d). At the latest follow-up, no new lesions were detected, and the patient remains under close surveillance (Figure 3).

## Discussion

The general terminology of BCC and treatment recommendations have been revised considerably within the recent National Comprehensive Cancer Network (NCCN) guidelines [5]. According to these guidelines, high-risk BCC is defined based on anatomic site, tumor size, histologic subtype, and clinical features. Facial H-zone lesions (such as periocular, perinasal, perioral, and auricular regions) are considered high-risk, whereas other facial sites, such as the cheek or forehead, are usually intermediate risk unless additional high-risk features are present. On the trunk and extremities, tumors ≥2 cm are regarded as high-risk, while on non-H-zone facial sites, tumors ≥1 cm may also fall into the high-risk category. Considering the recurrence risk in such cases, the traditional recommendation for surgical excision with a margin of 4-6 mm has been revised.

The NCCN 2024 guideline states: “Due to the wide variability of clinical characteristics that may define a high-risk tumor, it is not feasible to recommend a defined margin for standard excision of high-risk BCC. Keen awareness of the subclinical extension of BCC is advised when selecting a treatment modality without complete margin assessment for a high-risk tumor.” In addition to risk stratification, the potential functional and cosmetic consequences associated with facial surgical interventions should be carefully considered during treatment planning [5]. In recurrent and multifocal BCC, particularly in sun-exposed facial skin, the concept of field cancerization-where genetically altered keratinocytes predispose to new primary lesions-may also contribute to disease persistence and recurrence [9].

Vismodegib is an effective treatment option for advanced or recurrent BCC [7], especially when surgical interventions and radiotherapy cannot achieve clear margins. However, reaching optimal results may require long-term treatment. Although vismodegib is generally considered a safe medication that can be used in the geriatric population without serious adverse reactions or drug interactions, common side effects like taste disturbance and muscle cramps can reduce patient tolerance. Although topical agents such as imiquimod and 5-fluorouracil are frequently used for low-risk BCC lesions, a study by Bostancı et al. demonstrated their promising potential in different high-risk histological subtypes of facial BCC [10]. In patients undergoing multiple treatments and hesitant to receive further interventions, these topical agents can be used with meticulous dermoscopic follow-up.

Achieving negative surgical margins is critically important in the management of basal cell carcinoma (BCC) to minimize the risk of recurrence. Surgical excision should be performed with margins confirmed to be clear before proceeding with reconstructive procedures. Vismodegib has demonstrated efficacy in the treatment of recurrent BCC; however, its use may be constrained by adverse side effects, requiring a multidisciplinary approach that incorporates surgical intervention, systemic therapy, and topical treatments to optimize patient outcomes.

## Conclusions

Negative surgical margins must be ensured before reconstruction, and the management of challenging BCC often requires a multimodal approach combining surgical, systemic, and topical therapies.
